# An Insight on Current Clubfoot Management: A Reported Data From Lebanon

**DOI:** 10.7759/cureus.40194

**Published:** 2023-06-09

**Authors:** Zeinab Zeaiter, Hassan Alzein, Youssef Daher

**Affiliations:** 1 Department of Internal Medicine, Lebanese University Faculty of Medicine, Beirut, LBN; 2 Department of Orthopedics and Traumatology, Lebanese University Faculty of Medicine, Beirut, LBN; 3 Department of Orthopedic Surgery, Rafik Hariri University Hospital, Beirut, LBN

**Keywords:** relapse, bracing, abduction, ponseti’s technique, clubfoot

## Abstract

Background/aim: Clubfoot, which has been reported in the literature since the time of Hippocrates in 400 BC, is regarded as one of the most difficult congenital orthopedic anomalies, with a high relapse incidence of 16.87 infants per 10,000 births. The Lebanese region holds limited data concerning the evolution of clubfoot management. Herein, we aim to present novel findings in the treatment of clubfoot without surgical intervention.

Materials and methods: This single-center, cross-sectional research included 300 patients with virgin idiopathic clubfoot treated at our facility from 2015 to 2020. The Pirani and DiMeglio Scores were used to determine the severity of the illness prior to treatment, and the DiMeglio Score was used to determine the severity of the disease after treatment. For data analysis, the Statistical Package for Social Sciences (SPSS, IBM-Version 26; IBM Corp., Armonk, NY) was used and results with p-value < 0.05 were considered statistically significant.

Results: Our study included 300 patients, with 188 boys (62.7%) and 112 girls (37.3%). The mean age of the patients’ onset was 32 days. We recorded an average initial Pirani score of 4.27 ± 0.65 and an average initial DiMeglio score of 11.58 ± 2.56 (62 out of 300) while the average final DiMeglio score was 2.17 ± 1.82. The mean number of casts was 5 ± 0.8, with a minimum of four and a maximum of six casts. The prevalence of relapse was 20.7%.

Conclusions: Clubfoot remains a challenging deformity with a high rate of treatment failure and recurrence. While the superiority of Ponseti's technique in terms of success rate could not be disputed, tailored therapy based on the patient's socioeconomic status is considered critical for compliance and treatment success.

## Introduction

Hippocrates described clubfoot in 400 BC as one of the most prevalent congenital orthopedic anomalies. Remarkably, old drawings from Egyptian temples showed the progression of this deformity until adulthood in many individuals, such as Pharaon Siptah (12 BC) [1]. In his turn, Hippocrates advocated early therapy with serial bandages to correct foot anatomy [1-4].

Clubfoot is one of the most challenging congenital orthopedic anomalies. It affects 16.87 newborns per 10,000 births and has a high relapse rate regardless of the mean treatment (conservative vs. operative). Hence, it is considered to be one of the most common musculoskeletal deformities at birth [5]. The three-dimensional pathoanatomic components of this malformation are the equinus hindfoot varus, forefoot adductus, and midfoot cavus [6-8].

Diagnosis begins early during a routine intrauterine ultrasound in the second trimester and is clinically confirmed after birth [9]. Confirmation occurs through clinical assessment since radiographs are ineffective [7,9]. If untreated, the morbidity of this disease progresses into life-long deformity, disability, and stigma-related discrimination [10].

Treatments have remarkably evolved since Hippocrates's time, from simple orthopedic techniques to the development of Ponseti's technique. Manual manipulation is followed by successive casting with Achilles tendon tenotomy, which is then revealed by abduction. This technique has become the gold standard for treating congenital clubfoot all over the globe [11-14].

In times when the first antiquity reports emerged from the Middle East, limited data were available concerning the evolution of clubfoot management in our region. Smythe et al. estimated the incidence of clubfoot in Lebanon, Iraq, and Egypt to be 1.19 (0.98-1.40) per 1,000 live births in this area [15].

Lebanese data from the early 1980s revealed the adoption of Kite’s method as the first non-surgical treatment of clubfoot in Lebanon. This method was based on gentle adjustment through different steps: forefoot adduction manipulation, hindfoot varus correction through simple eversion of the calcaneus, and equinus midfoot varus correction. This indicates a possible casting of two years, thus limiting its adoption. This remains an advantage in a time when high failure rates, exceeding 50% of the cases, were recorded worldwide [16,17].

After the introduction of safe anesthesia techniques in newborns, Lebanon quickly became a leading nation in the Middle East for clubfoot surgical treatment. The local experience, combined with surgical reports, shifted the path of clubfoot therapy upon increased complications including ankle stiffness, subtalar joint arthritis, and residual deformity; consequently, leaving surgery behind [18,19].

In the early and mid-1990s, the calcaneopedal unit (CPU), Ponseti’s technique, and the French functional method were introduced in Lebanon for the treatment of congenital clubfoot [20,21]. Ponseti’s method became the gold standard treatment for clubfoot in Lebanon and the Middle East. Indeed, the French method required daily commitments for more than six months and showed possible residual equinus among patients [2,21].

Ponseti's method remains popular in the Middle East, but due to the ongoing economic crisis, it was difficult to implement it in Lebanon among patients with limited financial resources. Ponseti's technique necessitates weekly casts, which may require an Achilles tenotomy. In fact, when compared to high-income countries, a study on the clinical output and risk factors associated with Ponseti's technique in a low-resource environment showed a delay in serial casting, extended duration, and an elevated relapse rate.

In this study, we reviewed the records of patients who visited a Clubfoot facility in Beirut-Lebanon, between 2015 and 2020 and reported each patient’s characteristics (demographics and clinical), treatment patterns, and outcomes (relapse and complications). Descriptive statistics were presented for the different variables in addition to determining those that are associated with deformity relapse and that could also be considered risk factors.

## Materials and methods

Study design

Our research is a retrospective cross-sectional study conducted at a single center. From 2015 to 2020, 300 patients with virgin idiopathic clubfoot were enrolled as clubfoot patients who finished their casting and bracing protocol. This study was carried out in accordance with the Declaration of Helsinki (1964) and approved by the local ethics committee of the “Rafik HARIRI University Hospital” (Reference: 2023-0503). Written informed consent was obtained from the infants' parents.

Treatment modality

A treatment modality was attempted to be implemented based on patho-anatomy of the deformity that would be more practical and accessible to all patients in Lebanon. The first metatarsal was extended to correct the cavus malformation. To accomplish proper medial rotation of the navicular and cuboid bones in relation to the talus, serial abduction between 20° and 70° was performed by manipulating the talus. The most recent casting included 15-20° of dorsiflexion of the foot without Achilles tenotomy. The follow-up period lasted four years, with the first six months requiring Denice-Browne bar bracing for 23 hours. Bracing was restricted to bedtime by the sixth month.

Data collection

Severity scores were determined at the presentation using both the Dimeglio score DimS and Pirani score PirS [22,23]. Regarding DimS, midfoot rotation, hindfoot varus, forefoot adduction, and equinus, each was given 0-4 points based on reducibility on the relative plane. However, the pejorative items (posterior crease, medial crease, cavus, and muscular abnormality) were each scored as 1 if present and 0 if absent. The total of these elements is calculated on a 20-point scale, with a higher score indicating a more severe deformity.

For PirS, six different features of clubfoot deformity (posterior crease, emptiness of the heel, rigidity of the equinus, medial crease, curvature of the lateral border of the foot, and reducibility of the lateral part of the head of the talus) were evaluated. Each item was given a score of 0 (no abnormality), 0.5 (moderate abnormality), or 1.0 (severe abnormality) and summed to produce a final score between 0 and 6, where 6 is the most severe score. Sex, laterality (uni-/bilateral), family history, number of casts required for correction (excluding post-tenotomy cast), and the need for tenotomy were also analyzed.

Statistical analysis

Statistical Package for Social Sciences (SPSS, IBM-Version 26; IBM Corp., Armonk, NY) was used for data analysis. Descriptive analysis was used to represent the variables, in which nominal variables were represented by frequencies whilst continuous variables were represented by mean, median, standard deviation, minimum, and maximum.

Pearson Chi-square and unpaired Student’s t-test were performed to examine the factors associated with relapse. A binary logistic regression analysis was performed to determine relapse risk factors, and the model included all variables that were statistically associated with relapse. The odds ratios were presented with a 95% confidence interval. All results with p-values < 0.05 were considered statistically significant.

## Results

Study population

Demographic Characteristics of the Study Population

The demographic features of the studied population are shown in Table 1. The research study comprised 300 individuals, with 188 males (62.7%) and 112 girls (37.3%). Upon presentation, the average age was 32 days, with a median age of 21 days, a minimum of seven days, and a maximum of 100 days. The study comprised 170 Lebanese (56.7%), 71 Syrians (23.7%), 25 Palestinians (8.3%), and 34 patients of other nationalities (11.3%). Financial coverage varied among patients with 14.3% financially covered by Committee on National Security Systems (CNSS), 15% by the Ministry of Public Health (MOPH), and 14.3% by insurance, whereas the majority (56.3%) had no financial coverage (self-payment).

**Table 1:** Demographic characteristics of the study population (N = 300)

		Frequency	Percent
Sex	Boy	188	62.7
	Girl	112	37.3
Age at presentation (in days)	Mean (SD)	32.04 (26.22)	
	Median	21	
	Min - Max	7 - 100	
Nationality	Lebanese	170	56.7
	Syrian	71	23.7
	Palestinian	25	8.3
	Other	34	11.3
Financial Coverage	CNSS	43	14.3
	MOPH	45	15.0
	Insurance	43	14.3
	Self-Payer	169	56.3

Assessment of Clubfoot Deformity Different Features

Bilateral (42.3%) and unilateral (57.7%) clubfeet were seen in patients, as shown in Table 2. Moreover, 85% of the patients showed brace compliance, whilst no one had Achille tenotomies. The initial Pirani, initial DiMeglio, and final DiMeglio scores, that were used to evaluate each patient, had average values (Mean±SD) of 4.27±0.65, 11.58±2.56, and 2.17±1.82, respectively (Table 2).

**Table 2:** Clubfoot deformity features

		Frequency	Percent
Laterality	Unilateral	173	57.7
	Bilateral	127	42.3
Brace compliance	No	45	15.0
	Yes	255	85.0
Achille tenotomy	No	300	100.0
Initial Pirani score	Mean (SD)	4.27 (0.65)	
	Median	4	
	Min - Max	3 - 5.5	
Initial Dimeglio score	Mean (SD)	11.58 (2.56)	
	Median	12	
	Min - Max	5 - 17	
Number of casts	Mean (SD)	5.01 (0.66)	
	Median	5	
	Min - Max	4 - 6	
Final Dimeglio score	Mean (SD)	2.17 (1.82)	
	Median	2	
	Min - Max	0 - 7	

Clubfoot Deformity Relapse and Follow-up

Our data showed that 20.7% (62 patients out of 300) had clubfeet deformity relapse. Moreover, the average follow-up years was 3.18 ± 0.82 with a minimum follow-up of one year and a maximum follow-up of five years (Table 3).

**Table 3:** Relapse and follow-up

		Frequency	Percent
Relapse	No	238	79.3
	Yes	62	20.7
Follow-up years	Mean (SD)	3.18 (0.82)	
	Median	3	
	Min - Max	1 - 5	

Factors Associated With Clubfoot Deformity Relapse

In order to determine the variables that could be associated with deformity relapse, Pearson Chi-Square and unpaired Student’s t-tests were carried out. Results showed that girls were more likely to relapse than boys, according to an analysis of the demographic characteristics (p=0.043). In addition, a greater recurrence rate was linked to late presentation (p<0.001) (Table 4).

From an anatomical perspective, relapse was more common in unilateral instances (27.7%) than in bilateral cases (11%) (p<0.001). Relapsed patients had higher initial Pirani, initial DiMeglio, and final DiMeglio scores than those who did not (p 0.001). Brace compliance also evaded relapse (p<0.001) (Table 4).

The average number of casts was higher in patients who had relapse (p<0.001) (mean number of casts = 5.89 ± 0.41 among relapse patients vs. mean number of casts = 4.78 ± 0.50 for non-relapse patients). Additionally, patients who experienced relapse had longer follow up period (mean follow-up = 3.41-year vs. 2.31) (p<0.001) (Table 4).

**Table 4:** Factors associated with relapse Tests used are chi-square test (a) and unpaired student’s t test (b). Bold: statistically significant association set at 5%.

		Relapse		Total	P-value
		No	Yes		
Sex	Boy	156	32	188	0.043 ^a^
		83.0%	17.0%	62.7%	
	Girl	82	30	112	
		73.2%	26.8%	37.3%	
Nationality	Lebanese	133	37	170	0.082^ a^
		78.2%	21.8%	56.7%	
	Syrian	52	19	71	
		73.2%	26.8%	23.7%	
	Palestinian	24	1	25	
		96.0%	4.0%	8.3%	
	Other	29	5	34	
		85.3%	14.7%	11.3%	
Nationality	Lebanese	133	37	170	0.591^ a^
		78.2%	21.8%	56.7%	
	Other	105	25	130	
		80.8%	19.2%	43.3%	
Nationality	Syrian	52	19	71	0.147^ a^
		73.2%	26.8%	23.7%	
	Other	186	43	229	
		81.2%	18.8%	76.3%	
Financial Coverage	CNSS	37	6	43	0.355^ a^
		86.0%	14.0%	14.3%	
	MOPH	37	8	45	
		82.2%	17.8%	15.0%	
	Insurance	36	7	43	
		83.7%	16.3%	14.3%	
	Self-Payer	128	41	169	
		75.7%	24.3%	56.3%	
Unilateral or bilateral	Unilateral	125	48	173	<0.001^ a^
		72.3%	27.7%	57.7%	
	Bilateral	113	14	127	
		89.0%	11.0%	42.3%	
Brace compliance	No	4	41	45	<0.001^ a^
		8.9%	91.1%	15.0%	
	Yes	234	21	255	
		91.8%	8.2%	85.0%	
Age at presentation	Mean (SD)	24.38 (18.47)	61.45 (30.60)	32.04 (26.22)	<0.001^ b^
	Min - Max	7 - 100	7 - 100	7 - 100	
Initial pirani score	Mean (SD)	4.11 (0.55)	4.89 (0.64)	4.27 (0.65)	<0.001^ b^
	Min - Max	3.0 - 5.5	3.0 - 5.5	3.0 - 5.5	
Initial demeglio score	Mean (SD)	10.86 (2.26)	14.34 (1.57)	11.58 (2.56)	<0.001^ b^
	Min - Max	5 - 16	10 - 17	5 - 17	
Number of casts	Mean (SD)	4.78 (0.50)	5.89 (0.41)	5.01 (0.66)	<0.001^ b^
	Min - Max	4 - 6	4 - 6	4 - 6	
Final dimeglio score	Mean (SD)	1.36 (0.81)	5.27 (1.15)	2.17 (1.82)	<0.001^ b^
	Min - Max	0 - 4	1 - 7	0 - 7	
Follow-up years	Mean (SD)	3.41 (0.62)	2.31 (0.92)	3.18 (0.82)	<0.001^ b^
	Min - Max	1.0 - 5.0	1.0 - 4.0	1.0 - 5.0	

Binary Logistic Analysis for the Factors Affecting Relapse

In order to determine the variables influencing relapse, a binary logistic regression analysis was conducted. Results showed that the higher the DiMeglio score, the greater the likelihood of relapse (12, 95% CI [4.558 - 32.207] (p < 0.001)). On the other hand, brace compliance was a protective factor against relapse with an OR of 0.020 (95% CI [0.001 - 0.306] (p = 0.020)) (Table 5).

**Table 5:** Binary logistic analysis for the factors affecting relapse

	B	S.E.	p.value	OR	95% C.I.for EXP(B)	
					Lower	Upper
Final Dimeglio score	2.495	0.499	0.000	12.117	4.558	32.207
Brace compliance	-3.892	1.381	0.005	0.020	0.001	0.306
Constant	-5.824	1.767	0.001	0.003		

## Discussion

Our study data are intended to investigate the efficacy of an adapted way of treating clubfeet in Lebanon. Clubfoot was found to occur in 1.19 (0.98-1.40) per 1,000 live births in the Middle East region [24].

Until now, Ponseti’s technique has been the gold standard for treating clubfoot worldwide. This method requires serial manipulations and castings, Achilles tenotomy, and eventually abduction-bracing. A regional survey by Ghanem et al, assessing the approaches to clubfoot management by pediatric orthopaedical surgeons in the Middle East, showed a 97.1% adherence to Ponseti’s technique [25].

The economic situation in Lebanon has caused difficulties in all medical fields. Materials for orthopedic procedures are more expensive in a nation that relies on exports, putting them out of reach for all patients. Consequently, this delays therapy and reduces compliance with follow-up clinical visits. Furthermore, our study discovered that both the public and private insurance sectors provide insufficient coverage, with more than half of the population opting for self-pay. As an alternative to Ponseti's procedure, we treated our patients without the need for an Achilles tenotomy. Our study shows that clubfoot is more common in males than in females, which is consistent with previously reported data worldwide [26].

Assessing the severity of the disease, the average initial Pirani score was 4.27 ± 0.65, which is considerably low. The average initial DiMeglio score was 11.58 ± 2.56, which is considered less severe than the results reported in Italy [27]. The average final DiMeglio score was 2.17 ± 1.82. Unilateral cases were more common than bilateral cases (57.7% vs. 42.3%), which explains the lower initial Pirani and DiMeglio scores compared with international data. Indeed, bilateral cases are associated with higher initial Pirani and higher DiMeglio scores [27].

Bilateral deformity requires a longer treatment period, and more casting, and is associated with a higher relapse rate [27]. The mean number of casts was 5 ± 0.8, with a minimum of four casts and a maximum of six casts. The prevalence of relapse was 20.7% (62 of 300). The average years of follow-up was 3.18 ± 0.82 years, with a minimum follow-up of one year and a maximum follow-up of five years. According to bivariate analysis, the higher the DiMeglio score, the higher the risk of relapse (OR=12.117; 95% CI [4.558 - 32.207] (p<0.001)). Brace compliance is a protective factor to relapse with an OR = 0.020 (95% CI [0.001 - 0.306] (p = 0.020)). In fact, only 15% of our patients were noncompliant with bracing, which would have favored our approach's 80% success rate.

Previous data from a low resource center, where Ponseti’s technique was adopted, significantly showed that more abundant casts were needed among patients with bilateral foot deformities compared to those with unilateral deformity (bilateral 5.3 ± 1.7, unilateral 4.7 ± 1.7; p<0.011) [26].

In our population, the recurrence rate was higher than in Ponseti's technique. Wound infection (5.26%), followed by cast loosening (4.09%), was the most frequent complication in another study by Chotigavanichaya et al., where 15.2% needed a second operation for recurrent deformities [28].

Although our method is based on Ponseti’s technique, we did not perform Achilles tenotomy. A success rate of 80% is fair enough compared to the traditional Ponseti’s technique (90%-98% success rate) [29].

In a country threatened by continuous economic perturbations, adjusting guidelines to the population’s conditions is a reasonable approach to increasing success rates. In Lebanon, public transportation is chaotic, and since a significant portion of the population resides in rural regions, it may be difficult for parents to make weekly visits. Despite the fact that therapy effectiveness is dependent on compliance, it is critical to educate parents on the necessity of bracing. Indeed, we attempted to minimize exposure to surgery and encourage compliance with bracing, which was estimated to be 85%. Our study is a retrospective in which many values were missing. In addition, results may differ with time, and the recurrence rate may not be properly assessed in the future.

Although the sample size was adequate, multicentric data are required to confirm our findings. The most encountered biases were that virtually all individuals were treated, and we did not include a control population to compare our method to Ponseti's methods.

## Conclusions

Clubfoot is still regarded as a challenging deformity with a significant level of treatment failure and recurrence. The implementation of Ponseti's technique in Lebanon faces several obstacles, including the country's economic condition, limited hospital resources, inaccessible rural transportation, and the concentration of treatment institutions in the city of Beirut. It is essential to select an effective clubfoot treatment strategy that considers both success and compliance rates. Adopting Ponseti's concept and modifying it to limit surgical intervention would benefit patients, as our research found. In terms of success rate, we cannot dispute the superiority of Ponseti's technique. On the other hand, reducing the amount of weekly visits, improving compliance with bracing, and avoiding unnecessary surgeries would result in a significantly higher success rate.
